# Rituximab and lenalidomide combination treatment for rheumatoid arthritis complicated with myelodysplastic syndrome: A case report

**DOI:** 10.31138/mjr.28.4.217

**Published:** 2017-12-22

**Authors:** Gerasimos Evangelatos, Ioanna Vlachadami, Maria Kechagia, Alexios Iliopoulos

**Affiliations:** 1 Rheumatology Department, 417 Army Share Fund Hospital (NIMTS), Athens, Greece,; 2 Department of Pathophysiology, General Hospital of Athens “Laiko”, Athens, Greece

**Keywords:** rituximab, lenalidomide, rheumatoid arthritis, myelodysplastic syndrome

## Abstract

Rheumatoid arthritis patients might experience several hematologic complications. The development of myelodysplastic syndrome is not clearly associated with RA, even though it has been described in some patients with pre-existing disease. There are only few data available in the literature concerning the therapeutic approach of such patients. Herein, we report a case of RA complicated with progressive MDS, successfully treated with rituximab and lenalidomide combination therapy.

## INTRODUCTION

Rheumatoid arthritis (RA) is an autoimmune disease of joints. Patients with RA are under a state of chronic systemic inflammation, sometimes leading to several complications,^1^ such as solid tumors and blood dyscrasias. Common hematologic manifestations in RA include anemia and thrombocytosis; whereas, in rare cases thrombocytopenia, eosinophilia and Felty’s Syndrome have been reported. Some patients might also present a marginally increased risk of developing lymphoma. Myelodysplastic syndromes (MDS) are a group of hematologic disorders characterized by abnormal blood cell maturation in the bone marrow. The association between RA and MDS has not been clearly defined. In a study of 68 patients with inflammatory arthritis and MDS, arthritis preceded MDS in 12 cases (55%), the diagnosis of both diseases was concomitant in 6 cases (27%) and arthritis occurred after MDS in 4 (18%).^2^ In a large cohort of 1408 MDS patients, 28% (391) had an autoimmune disease, and in 10% (41) of these cases, this disease was RA.^3^ There is data supporting that RA patients have 52% (OR 1.52) increased risk to develop MDS compared to healthy controls;^4^ however, some researchers claim that this slightly increased risk might derive from misdiagnosing early MDS symptoms similar to those of autoimmune disorders.^5^ In any case, the treatment of RA patients with co-existent MDS is a clinical challenge in rheumatology departments. We present a case of a patient with a long-standing history of RA that developed severe MDS, successfully treated with rituximab (RTX) and lenalidomide (LEN) combination therapy, resulting in remission of both diseases.

## CASE REPORT

A 66-year-old female patient with a 13-year history of seropositive RA developed a progressively deteriorating macrocytic hyperchromic anemia. The patient was treated with subcutaneous adalimumab (ADA) 40mg per 14 days and per os methotrexate (MTX) 10mg per week, in addition of folic acid 5mg per week. Vitamin B12 and folic acid levels were within normal range, while erythropoietin levels were elevated. Alcohol abuse was excluded. Although MTX was discontinued in order to eliminate drug-induced aplastic anemia from the differential diagnosis, hemoglobin (Hb) levels did not change. A bone marrow biopsy was performed, revealing dysplastic changes of erythrocyte lineage, which were characterized as reacting to patient’s autoimmune disorder.

**Figure 1.**
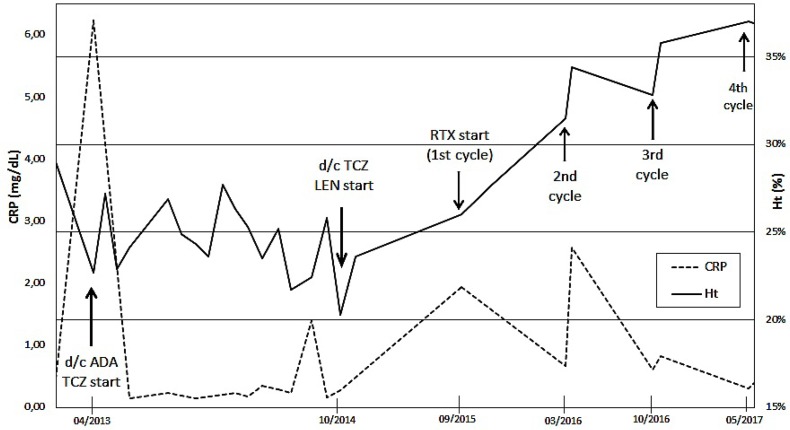
Hematocrit (Ht) and C-reactive protein (CRP) levels are demonstrated in correlation with time (patient’s visits at our department for the last 4 years). Secondary failure of adalimumab (ADA) led to treatment switch to tocilizumab (TCZ). Secondary failure of TCZ, in the context of progressively deteriorating anemia, resulted in TCZ discontinuation and initiation of lenalidomide (LEN). Following eleven months of LEN monotherapy, rituximab (RTX) treatment was initiated due to an arthritis flare. After four cycles (2 years) of RTX and LEN combination therapy, both anemia and joint inflammation are controlled.

The patient was experiencing high disease activity and anemia deterioration, even though on treatment with ADA. Therefore, a treatment switch to intravenous (i.v.) tocilizumab (TCZ) 8mg/kg every 4 weeks followed. This resulted to clinical remission of patient’s arthritis and significant decrease of ESR and CRP levels. On the contrary, the patient’s anemia continued to deteriorate gradually, and she became blood transfusion-dependent. Thrombocytosis was also observed and remained stable despite the treatment change, with platelet levels varying between 495,000 and 747,000/μL. After evaluation by a hematologist, karyotype examination was conducted to examine possible cytogenetic abnormalities. 5q deletion (5q-) syndrome was identified and therapy with per os LEN was initiated. LEN was administered in monthly cycles, continuously from Day 1 to Day 21, with a drug-holiday between Day 22 and Day 28. TCZ therapy was discontinued due to a secondary failure to control RA activity and to a suspected relation with transient pancytopenia, developed soon after initiation of LEN therapy. Following ten months of LEN monotherapy, hemoglobin levels increased from 6.7 g/dL to 8.1 g/dL, however, RA flared-up (DAS28-ESR=5.6, ESR= 92 mm/1^st^ hour, CRP= 1.46 mg/dL) leading in a worsening of the quality of life. We therefore, considered RTX as a therapeutic option for RA with a possible favorable effect to MDS as well. RTX was administered in dosages indicated for RA (i.v. cycles of 1g on Day 1 and Day 15, every 6 months), concomitantly with LEN. Following the first cycle of RTX-LEN combination, the patient’s arthritis presented a satisfactory clinical response, as well as a significant improvement in ESR, CRP and full blood count levels. Two years later (4 treatment cycles of RTX), hemoglobin, hematocrit and platelet (PLT) levels are within normal range (Hb= 12.3 g/dL, Ht= 37.0%, PLT= 182,000/μL) and joint inflammation is characterized by sustained clinical and laboratory remission (DAS28-ESR= 1.78, ESR= 7 mm/1st hour, CRP= 0.39 mg/dL). The patient currently remains under treatment with LEN. In **Figure 1**, the levels of hematocrit and C-reactive protein are illustrated in relation to every therapeutic regimen.

## DISCUSSION

To our knowledge, this is the first case to report that a biologic DMARD combined with other immunomodulatory drug resulted in remission of both RA and MDS. Tsuji et al. reported a case of RA-related MDS treated with cyclosporine.^6^ Moreover, Giagounidis et al. presented a case of RA complicated with 5q- myelodysplastic syndrome, which was treated with cytarabine.^7^ In this case, RA was treated only with steroids and cytarabine did not have any impact on arthritis activity.

RTX is a second line biological DMARD treatment choice for patients with RA according to ACR 2015 and EULAR 2016 guidelines, well-established for its efficacy and safety profile.^8^ RTX is approved for use after TNF-inhibitors failure or in case of other biological agents are contraindicated such as a history of lymphoma or demyelinating disorders, based on its efficacy in these diseases.^9,10^ In comparison to other biologic agents, RTX carries an increased risk of HBV re-activation.^11^ Thus, screening for HBV and HCV should be performed in every patient prior to RTX initiation.^12^ RTX use in RA patients has not been associated with any change in hemoglobin levels, although anemia has been reported to occur in 1.1 % of hematological patients treated with RTX.^13^ A low incidence of early or late onset neutropenia has been reported in these patients.^14^ With regard to the use of RTX in MDS patients, it has only been reported that RTX might reduce platelet transfusion refractoriness induced by multiple blood transfusions.^15,16^ RTX has been already used effectively in other hematologic conditions, such as autoimmune hemolytic anemia and immune thrombocytopenia.^17,18^ Interestingly, RTX has been successfully used in a case of MDS following systemic lupus erythematosus, leading to transfusion independence.^19^

LEN, a thalidomide analog, is being used to treat multiple myeloma and MDS, especially in patients with chromosome 5q deletion syndrome, by favoring apoptosis in del(5q) cells and suppressing p53 levels in erythroid progenitor cells.^20^ LEN seems to suppress TNF pathway genes expression^21^ and, recently, a study reported that LEN has a positive impact on type II collagen-induced arthritis model in rats.^22^ In our case, however, LEN monotherapy did not influence RA activity.

LEN plus RTX combination has been previously used in the treatment of Mantle-Cell Lymphoma, recurrent Follicular Lymphoma and indolent Non-Hodgkin Lymphoma with an accepted safety profile. In these cases, RTX was used in doses of 375 mg per square meter of body-surface area every 4 weeks, which differs from the dosage scheme of RA. It has been reported that LEN might enhance RTX-mediated B cell depletion through antibody-dependent cellular cytotoxicity.^23^ The main adverse event of LEN plus RTX combination was myelosuppression. LEN monotherapy has been also associated with venous thrombosis. In our case, following 2 years of RTX and LEN combination therapy, the patient remains free of serious infections and never required treatment with antibiotics. She has also experienced neither neutropenia, nor thrombocytopenia or any thromboembolic event.

Therapy-induced MDS due to methotrexate and leflunomide has been reported in RA patients,^24^ however, our patient’s MDS could not be attributed neither to methotrexate nor to adalimumab. The patient was under folic acid supplementation, folic acid blood levels were within normal range and two years after MTX discontinuation the hemoglobin levels continued to worsen. A recent study showed that patients under treatment with anti-TNF agents do not have an increased risk for MDS.^25^ Detailed literature search did not reveal any association between 5q deletion and methotrexate or TNF-a inhibitors.

Finally, despite the fact that the presence of RA and MDS in the same patient could be an incidental co-existence, further investigation might elucidate the possible role of B cells in RA-associated MDS.

In conclusion, the combination of RTX and LEN that was used in our patient resulted in sustained remission of both diseases, regarding clinical and hematological parameters. We, therefore, suggest that 5q deletion should be sought in patients with RA and MDS, and in this case, the combination of RTX and LEN seems to be a reasonable treatment option.
